# The Tumor Suppressor *Adenomatous Polyposis Coli (apc)* Is Required for Neural Crest-Dependent Craniofacial Development in Zebrafish

**DOI:** 10.3390/jdb11030029

**Published:** 2023-06-29

**Authors:** Xiaolei Liu, William D. Jones, Mathieu Quesnel-Vallières, Sudhish A. Devadiga, Kristin Lorent, Alexander J. Valvezan, Rebecca L. Myers, Ning Li, Christopher J. Lengner, Yoseph Barash, Michael Pack, Peter S. Klein

**Affiliations:** 1 Department of Medicine (Hematology-Oncology), Perelman School of Medicine, University of Pennsylvania, Philadelphia, PA 19104, USA; 2 Cell and Molecular Biology Graduate Group, Perelman School of Medicine, University of Pennsylvania, Philadelphia, PA 19104, USA; 3 Department of Genetics, Perelman School of Medicine, University of Pennsylvania, Philadelphia, PA 19104, USA; 4 Faculty of Arts and Sciences, University of Pennsylvania, Philadelphia, PA 19104, USA; 5 Department of Medicine (Gastroenterology), Perelman School of Medicine, University of Pennsylvania, Philadelphia, PA 19104, USA; 6 Department of Biomedical Sciences, School of Veterinary Medicine, University of Pennsylvania, Philadelphia, PA 19104, USA; 7 Department of Cell and Developmental Biology, Perelman School of Medicine, Institute for Regenerative Medicine, University of Pennsylvania, Philadelphia, PA 19104, USA

**Keywords:** APC, GSK-3, mTOR, cranial neural crest, EMT, splicing, complement C3, craniofacial, pharyngeal arch, Wnt, colon cancer

## Abstract

Neural crest (NC) is a unique vertebrate cell type arising from the border of the neural plate and epidermis that gives rise to diverse tissues along the entire body axis. Roberto Mayor and colleagues have made major contributions to our understanding of NC induction, delamination, and migration. We report that a truncating mutation of the classical tumor suppressor *Adenomatous Polyposis Coli* (*apc)* disrupts craniofacial development in zebrafish larvae, with a marked reduction in the cranial neural crest (CNC) cells that contribute to mandibular and hyoid pharyngeal arches. While the mechanism is not yet clear, the altered expression of signaling molecules that guide CNC migration could underlie this phenotype. For example, *apc^mcr/mcr^* larvae express substantially higher levels of *complement c3*, which Mayor and colleagues showed impairs CNC cell migration when overexpressed. However, we also observe reduction in *stroma-derived factor 1* (*sdf1/cxcl12*), which is required for CNC migration into the head. Consistent with our previous work showing that APC directly enhances the activity of glycogen synthase kinase 3 (GSK-3) and, independently, that GSK-3 phosphorylates multiple core mRNA splicing factors, we identify 340 mRNA splicing variations in *apc* mutant zebrafish, including a splice variant that deletes a conserved domain in *semaphorin 3f* (*sema3f*), an axonal guidance molecule and a known regulator of CNC migration. Here, we discuss potential roles for *apc* in CNC development in the context of some of the seminal findings of Mayor and colleagues.

## 1. Introduction

The neural crest (NC) is a remarkable vertebrate cell type induced at the border of the neural plate and adjacent epidermis. NC has the potential to develop into diverse cell types including muscle, cartilage, bone, peripheral and enteric neurons, glia, endocrine cells, melanocytes, and portions of the cardiac outflow tract, aortic arch, and semilunar valves [1,2,3,4,5,6]. The cranial neural crest (CNC) arises from the anterior neural plate border and gives rise to craniofacial muscles, cartilage, and bones as well as the cephalic peripheral nervous system, and thereby, gives rise to much of the vertebrate head and face.

Roberto Mayor and his colleagues have made major contributions to the study of NC induction, delamination, and migration and the cell biology and biomechanics of migration after NC cells leave the neural folds and move toward their diverse destinations [1,7,8,9,10,11,12,13,14,15,16,17,18]. As the neural tube closes, NC cells undergo an epithelial to mesenchymal transition (EMT), delaminate, and begin their migration throughout the body. CNC cells initiate migration as a unified group of cells, whereas trunk NC cells delaminate individually [19]. The complex sequence of instructions required for NC to undergo EMT, migration, and the eventual invasion of distant points has often been compared to the metastasis of cancer cells [4,20]. Mayor and colleagues have been pioneers in using the delamination of the NC for the in vivo study of EMT under physiological conditions.

The Mayor group has shown that, in the early phase of NC migration, cells maintain contact through several mechanisms. A seminal paper by Carmona-Fontaine et al. [10] defined an essential role for the contact inhibition of locomotion (CIL) in NC migration. CIL had been described previously as a cell behavior in which cells reorient their direction of migration upon contact with another cell [21]. Mayor, Stern, and colleagues showed that NC cells from *Xenopus laevis* and zebrafish depend on CIL for oriented migration; when a NC cell encounters another NC cell, it stops and reorients its direction of migration along with other NC cells. In contrast, when it encounters a different cell type, it invades that other tissue, as they showed using NC explants as well as in vivo imaging [10]. Within a migrating group of cells, the cells at the leading edge maintain greater polarity than internal cells and appear to lead directed movement, while the internal cells are primarily attracted to each other. The CNC cells of *Xenopus* secrete the complement factor C3, which is cleaved to generate the chemoattractive C3a polypeptide. CNC cells also express the C3a receptor [11], allowing mutual attraction, termed by the Mayor group as co-attraction (CoA). Furthermore, the overexpression of C3a diverts CNC from their normal path and impairs collective CNC cell migration, reinforcing the conclusion that C3a maintains CNC as a coherent group of cells to allow collective cell migration [11,17].

Wnt signaling plays essential and complex roles in NC development. Canonical Wnt/β-catenin signaling induces NC beginning in the gastrula stage, but this pathway must also be inhibited for the delamination of NC [13,22]. The secreted Wnt pathway inhibitor Draxin also modulates the timing of CNC EMT and migration [23]. Canonical Wnt signaling is also reduced in migrating CNC, and the ectopic activation of canonical Wnt signaling inhibits CNC cell migration [24]. Once CNC cells have arrived at their target tissues, the activation of canonical Wnt signaling promotes the differentiation of CNC, for example into chondrocytes [25].

While exploring the in vivo interplay between two Wnt pathway suppressors, Adenomatous Polyposis Coli (APC) and Glycogen Synthase Kinase 3 (GSK-3) [26,27,28], we uncovered a striking defect in the CNC contribution to craniofacial structures in *apc* mutant zebrafish. As presented here, this CNC defect is associated with marked elevation in *c3* expression; one hypothesis to explain this defect, therefore, is that elevated *c3* at an earlier stage of development in *apc* mutants disrupts the migration of CNC into the head, similar to the findings of Carmona-Fontaine at earlier stages of CNC migration [11]. *APC* is a classical tumor suppressor gene and GSK-3 is a protein kinase that phosphorylates the canonical Wnt signaling effector β-catenin. APC and GSK-3 function together to suppress the canonical Wnt signaling pathway by promoting the degradation of β-catenin. All of these proteins bind to the scaffold protein Axin, where APC directly enhances GSK-3 enzymatic activity toward β-catenin [28]. Wnt signaling is activated when an extracellular Wnt protein binds to a receptor complex and promotes the dissociation of APC from the Axin complex [28,29,30], leading to reduced GSK-3 enzymatic activity in the absence of APC.

To characterize the APC-dependent regulation of GSK-3 in more depth in vivo, we have examined the zebrafish *apc^mcr^* mutant [26], which has a truncating mutation in the mutation cluster region (mcr) that is similar to mutations associated with human colon cancers. The zebrafish *apc^mcr/mcr^* mutation is lethal in the early larva and causes multiple developmental phenotypes, including defects in heart looping, the expansion of endocardial cushions, reduced blood flow, liver hyperplasia, and the disruption of the anterior-posterior body axis [31,32]. These phenotypes are due to reduced Gsk-3 activity in the absence of full-length Apc. As a major role for GSK-3 in the Wnt pathway is to phosphorylate β-catenin and target it for degradation, some of the *apc* phenotypes arise through the accumulation of β-catenin [31]. However, several major phenotypes in *apc^mcr^* zebrafish are caused by the activation of other GSK-3 targets, including the nutrient sensor mechanistic target of rapamycin complex 1 (mTORC1) [26].

While examining Apc- and Gsk-3-dependent phenotypes in *apc^mcr^* mutants, we observed a highly penetrant defect that results in the accumulation of disorganized tissue just anterior to the developing heart. The examination of a CNC reporter shows marked reduction in the CNC contribution to craniofacial structures derived from the first and second pharyngeal arches along with the expansion and disorganization of CNC in more posterior pharyngeal arches. The transcriptomic comparisons of wild-type and *apc* mutant fish demonstrate the robust activation of canonical Wnt signaling, as expected, but also show the marked elevation of Complement Factor *c3* expression, which could contribute to this aberrant CNC phenotype by interfering with or diverting the migration of CNC that normally populates the first and second pharyngeal arches. However, we also observe changes in the expression of the other regulators of both differentiation and migration that may also play a role in the CNC phenotype we report here. Because GSK-3 is a novel global regulator of mRNA splicing, we also examined splicing in *apc* mutant zebrafish and identified hundreds of alternative spliced mRNAs, affecting a preponderance of mRNAs that regulate cell migration, specifically in the *apc* mutants.

## 2. Methods

### 2.1. Zebrafish Husbandry and Methods

Embryos were raised at 28.5 °C in standard E3 medium [26,33]. *apc^mcr^* zebrafish (kindly provided by Dr. Adam Hurlstone) and primers used for genotyping were described previously [26]. The live imaging of *fli1-GFP* larvae was performed with an Olympus MVX10 fluorescent dissecting microscope (Tokyo, Japan). The confocal imaging of *fli1-GFP* larvae was performed on a Zeiss LSM 510 confocal microscope. In situ hybridization was performed on embryos and larvae fixed at the stages indicated in the figures according to well-described methods [34]. Probes included *gsc*, *crestin*, *sox10*. Plasmids for *gsc* and *crestin* were kindly provided by Dr. Mary Mullins (University of Pennsylvania School of Medicine). For *sox10* in situ hybridization, an 832 bp fragment of *sox10* was PCR amplified with the primers GTCACTAAAGGTCCAACCGT and TGTGATGGACTTGAGGCACT and the product was subcloned into pCRII-TOPO; this plasmid was used as a template for the in vitro transcription of in situ hybridization probes. Zebrafish husbandry and egg procurement were carried out in accordance with the guidelines of the University of Pennsylvania Institutional Animal Care and Use Committee.

### 2.2. RNA Isolation, RT-PCR, and Sequencing

*apc^mcr/mcr^* homozygous embryos were sorted from wild-type and *apc^mcr/+^* heterozygotes based on morphology (homozygotes displayed a bent A–P axes, whereas heterozygotes were morphologically indistinguishable from WT) at 24 h post-fertilization (hpf) and harvested for RNA isolation at 48 hpf. A total of 20 embryos per genotype were collected from 3 independent clutches, and RNA was isolated using Trizol reagent, as described previously [35]. Genomic DNA was removed via DNAse treatment and total RNA was recovered using an RNeasy kit (Qiagen, Germantown, MD, USA). Purity was confirmed via a bioanalyzer (RIN > 8). RNAs were sent to Genewiz for polyA-selection, for the generation of stranded Illumina RNA-Seq library, and for sequencing at a depth of ≥50 million reads per sample replicate. For standard qRT-PCR, 50 ng RNA per sample was used for first-strand cDNA synthesis using SuperScript-III reverse transcriptase (Invitrogen, Waltham, MA, USA), according to manufacturer’s protocol. Relative gene expression was quantified by real-time PCR using Power Sybr Green PCR Master Mix, as described [36]. GAPDH was used as a control for RNA input. PCR primer sequences are shown in Table 1.

### 2.3. Quantification of Alternative Spliced and Differentially Expressed Genes

Alternative splicing and differential expression analyses were based on RNA-Seq reads that were mapped to the zebrafish reference genome (GRCz10) using STAR (https://www.ncbi.nlm.nih.gov/pmc/articles/PMC3530905/, accessed on 15 January 2018). Differential splicing analysis was performed comparing WT/*het* and *apc^mcr/mcr^* samples using Modeling Alternative Junction Inclusion Quantification (MAJIQ) deltapsi with intron retention quantification enabled. Results were filtered for high confidence changing local splice variants (LSVs) with a change in percent spliced in (dPSI) ≥20% (in which junctions had a ≥90% probability of expected dPSI).

### 2.4. Statistical Analysis and Downstream Bioinformatics

The results are presented as mean ± SD. Unpaired two-tailed Student’s *t*-tests were applied RT-qPCR data to determine differences in mRNA expression. *p* values were calculated following log_2_ transformation. Gene expression data from RNA-seq were obtained by aligning poly-A reads to the zebrafish reference genome (GRCz10) using STAR as described previously [35]. The generation of heatmaps and clustering analyses were performed using Morpheus (https://software.broadinstitute.org/morpheus/, accessed 22 March 2019). GO and KEGG (Kyoto Encyclopedia of Genes and Genomes) pathway analysis were performed using Metascape [37]. The list of genes that changed ≥2-fold with FDR <0.05 in *apc^mcr/mcr^* mutants vs. combined WT and *apc^mcr/+^* heterozygotes was submitted as the gene list. For Gene Set Enrichment Analysis (GSEA), Zebrafish Information Network (ZFIN) gene IDs were converted to homologous human gene symbols in Ensembl version 95 using the Bioconductor biomaRt package for Ensembl BioMart [38,39,40] and this list was submitted to GSEA 4.3.2 (https://www.gsea-msigdb.org/gsea/index.jsp, accessed 26 November 2022) to determine enriched pathways [41,42] Supplemental Table S3 shows GSEA identified Hallmark pathways ranked by normalized enrichment score.

## 3. Results

### 3.1. The Tumor Suppressor Apc Is Required for the CNC Contribution to Craniofacial Structures

**Figure 1:**
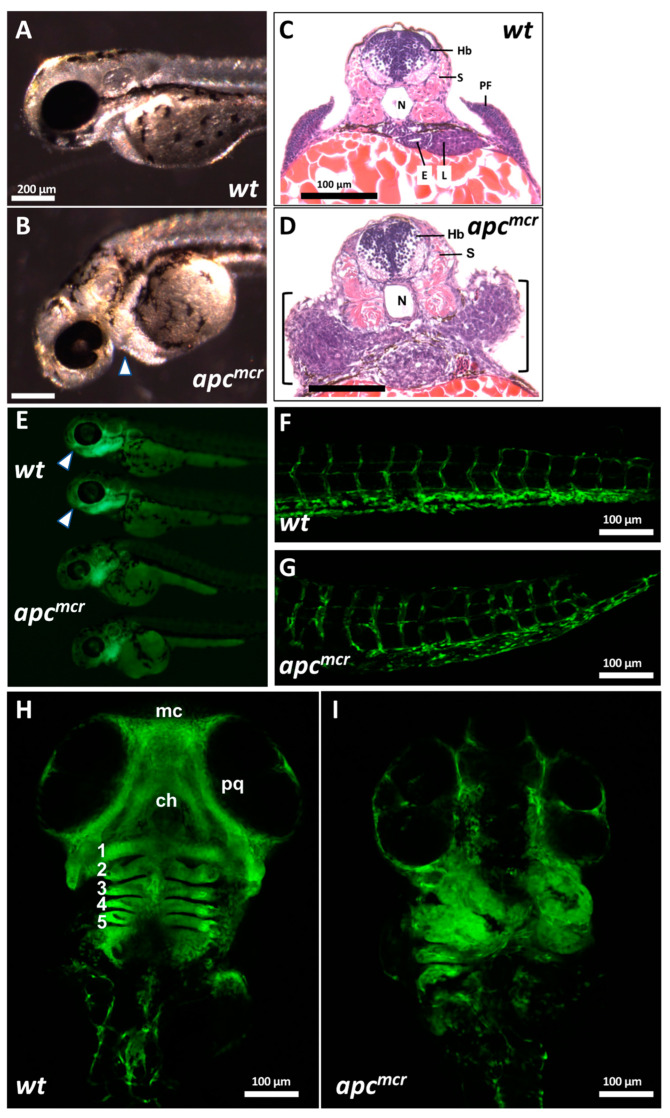
*apc* is required for the CNC contribution to craniofacial structures: (**A**) Wild-type (WT) and (**B**) *apc^mcr/mcr^* larvae at ~30 somites (50 hpf). Arrowhead indicates the accumulation of amorphous tissue anterior to the heart and yolk mass. Scale bars in (**A**,**B**) are 200 µm. (**C**,**D**) Hematoxylin-eosin staining of WT (**C**) and *apc^mcr/mcr^* (**D**) larvae sectioned at the level of the hindbrain. Hb: hindbrain; S: somite; N: notochord; E: esophagus; L: liver; PF: pectoral fin. Scale bars in (**C**,**D**) are 100 µm. (**E**) Fli1-GFP epifluorescence of WT (top two larvae) and *apc^mcr/mcr^* larvae (lower two larvae; 30-somite stage). Arrowheads for WT larvae indicate anterior extent of GFP^+^ cells in the head, which is reduced or absent in mutants. (**F**,**G**) Confocal image of WT; *Fli1-GFP* (**F**) and *apc^mcr/mcr^*; *Fli1-GFP* (**G**) larvae at 60 hpf, showing the vascular endothelium of trunk. (**H**,**I**) Confocal image of Fli1-GFP in WT (**H**) and *apc^mcr/mcr^* (**I**) larvae at 60 hpf, showing CNC and vascular endothelium in branchial arches and head. mc: Meckel’s cartilage; pq: palatoquadrate cartilage; ch: ceratohyal cartilage. Numbers indicate gill arches 1–5.

Defects in morphogenesis leading to curvature of the anterior–posterior axis are a prominent feature of the *apc^mcr^* mutant [26,31]. This phenotype is detectable as early as 24 hpf, and by 50 hpf (~30 somites), it is associated with a collection of amorphous tissue anterior to the yolk mass and developing heart (Figure 1A–D). As the identity of the cells contributing to this aberrant tissue mass was not clear, we crossed *apc^mcr^* mutants with reporter lines that express GFP in vascular endothelial cells (*fli1-GFP*) [43], renal tubular epithelium (*enpep-GFP*), gut endoderm (*foxa3-GFP*), and smooth muscle (*sm22alpha-GFP*). Although most tissue-specific markers showed relatively normal expression patterns within the region of interest, *fli1-GFP*; *apc^mcr^* mutant larvae showed a high accumulation of GFP^+^ cells within these amorphic cell clusters and markedly reduced GFP^+^ cells in the ventral head (Figure 1E). *fli1-GFP* is expressed in vascular endothelium throughout the larva and in CNC-derived pharyngeal arch mesenchyme [43]. Confocal imaging showed that *Fli1-GFP^+^* expression is similar in the vascular structures of the trunk in wild-type (WT) and *apc^mcr^* mutants (Figure 1F,G). In stark contrast, GFP^+^ cells that are prominent in craniofacial structures derived from the first and second pharyngeal arches in wild-type larvae, including Meckel’s cartilage, the palatoquadrate cartilage, and the ceratohyal cartilage (Figure 1H), are markedly reduced or absent in *apc* mutants. These reductions are accompanied by an increased number and disorganization of Fli1-GFP^+^ cells within more posterior pharyngeal arches (Figure 1I), corresponding to the aberrant cell cluster we observe in intact larvae and by histological sectioning (Figure 1B,D). The low level of GFP^+^ cells present in the heads of *apc^mcr^* mutants may reflect reduced vascular endothelial and/or CNC cells.

**Figure 2:**
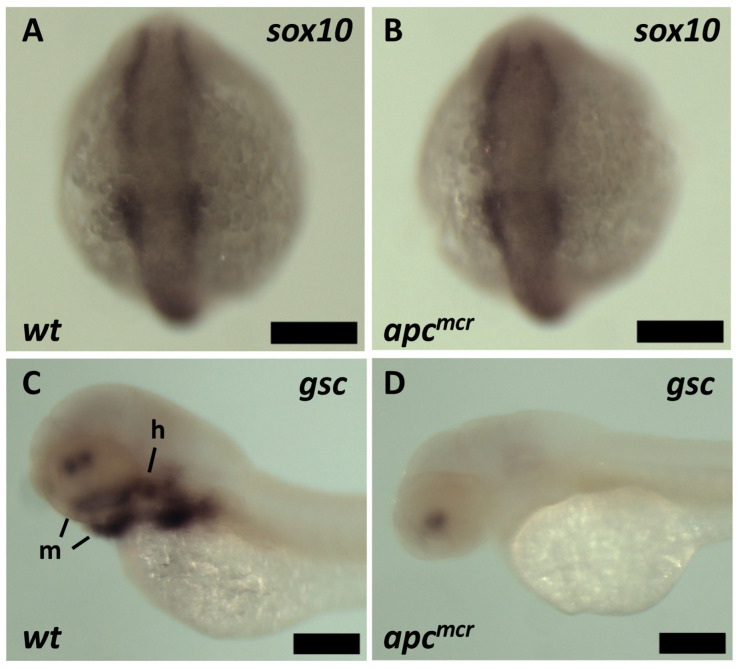
Expression of early and late neural crest markers by in situ hybridization: (**A**,**B**) Similar *sox10* expression at neural plate border in WT (**A**) and *apc^mcr/mcr^* (**B**) embryos at 12 hpf. Genotypes in (**A**) (*n* = 30 WT or *apc^mcr/+^*) and (**B**) (*n* = 7 *apc^mcr/mcr^*) were defined by genomic PCR on individual larvae. (**C**,**D**) In WT embryos at 55 hpf (**C**), *gsc* is expressed in the mandibular (m, including Meckel’s cartilage), hyoid (h), and more posterior branchial arches, as well as two symmetric anterior domains, as described previously [44]. In *apc^mcr/mcr^* larvae (**D**), *gsc* is undetectable in the mandibular and hyoid arches. (For WT, *n* = 15, and for *apc^mcr/mcr^*, *n* = 7, based on morphological phenotype). All scale bars are 200 µm.

**Figure 3:**
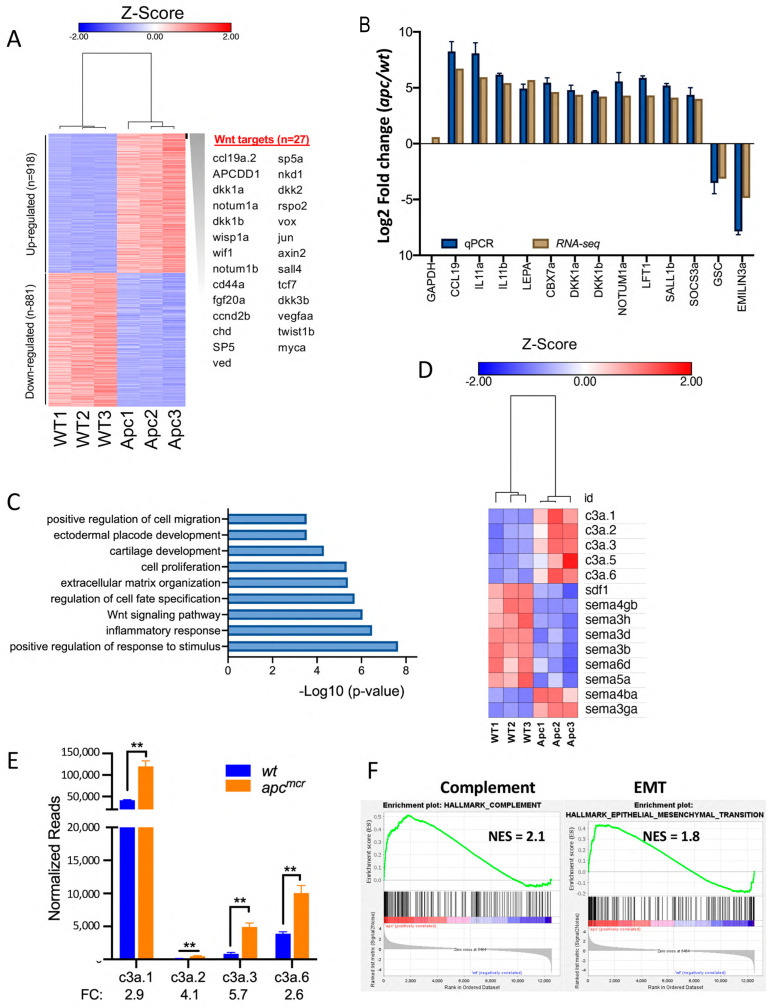
Transcriptomic analysis of *apc* loss of function larvae: (**A**) Heat map showing differential gene expression in WT versus *apc^mcr/mcr^* larvae at 48 hpf. To the right is a list of a upregulated genes known to be induced by Wnt/β-catenin activation [45], including multiple Wnt-induced pathway antagonists. (**B**) RT-qPCR was used to validate changes in expression from RNA-seq data for the indicated mRNAs. GAPDH was used as an input control for RT-qPCR. Error bars show standard error. (**C**) GO analysis (Metascape [37]) shows the differential expression of genes associated with the indicated terms in *apc^mcr^* mutants. (**D**) Heat map showing the differential expression of mRNAs encoding secreted molecules that regulate neural crest migration and axon guidance. In zebrafish, Complement *c3* is encoded by “a” and “b” alleles and the *c3a* alleles were duplicated to six copies, *c3a.1–c3a.6*. The “*c3a*.x” designation for these duplicated genes should be distinguished from the more common designation of the polypeptide “C3a”, the extracellular chemoattractant derived via the proteolytic processing of C3 protein that mediates the co-attraction of CNC cells [11]. The heat map also shows the reduced expression of *SDF1/CXCL12* and the differential expression of multiple semaphorins. (**E**) The abundance of *c3a.1*, *c3a.2*, *c3a.3,* and *c3a.6* genes in WT and *apc^mcr^* larvae at 48 hpf was based on mean normalized read counts from three independent replicates from RNA-seq data. c3a.5 was excluded because mean read counts were <100 and c3a.4 was not detected. Fold change in expression for *apc^mcr^*/WT is shown below graph. Relative change in expression was confirmed for c3a.1 and c3a.6 by RT-qPCR (not shown). Error bars show standard error. ** indicates *p* < 0.01 (Student’s *t*-test). (**F**) GSEA analysis [41] for *apc^mcr^*/WT zebrafish identifies parallels with the complement pathway and EMT, as well as c-Myc, canonical Wnt signaling, mTORC1 activation, and inflammatory signaling pathways (Supplemental Table S3).

The absence of CNC cells in the derivatives of the mandibular and hyoid arches could reflect the disruption of CNC specification or migration. However, this defect is not caused by a disruption of initial NC induction, as the expression of the early NC marker *sox10* at the neural plate border is normal in *apc* mutants at the 5-somite stage (12 hpf; Figure 2A,B). The overall level of expression of the pan-NC marker *crestin* was also similar in WT and *apc^mcr^* larvae at 33 hpf. However, at later stages of larval development (55 hpf), the paired-like homeobox transcription factor *goosecoid (gsc),* which is highly expressed in the mandibular, hyoid, and more posterior pharyngeal arches in WT larvae (Figure 2C, [44]), was absent from these CNC-derived structures in *apc^mcr^* mutant larvae (Figure 2D). The reduced expression of *gsc* in *apc^mcr^* mutants was also observed via RNA-seq, as described below (Figure 3). These findings demonstrate a marked defect in craniofacial development in *apc^mcr^* mutants that we hypothesize was caused by a failure of CNC to migrate into the head, although, alternatively, altered cell fate specification could still underlie this defect as well.

### 3.2. Increased Expression of Cytokines and Wnt Target Genes in apc^mcr/mcr^ Larvae

To gain a deeper understanding of how Apc regulates the expression of genes that may influence CNC specification and/or migration, as well as more general insights into the role of APC in global gene expression, we performed RNA sequencing (RNA-seq) on the wild-type and *apc* mutant larvae at 48 hpf. RNA-seq was performed with three biological replicates and sequenced at a depth of ≥50 million reads per sample. Using a threshold of ≥ 2-fold change and false discovery rate (FDR) < 0.05, we observed the increased abundance of 918 mRNAs and the decreased abundance of 881 mRNAs in the *apc^mcr^* mutant compared to wild-type larvae (Figure 3A and Supplemental Table S1).

The expression of >30 established Wnt target genes and Wnt-inducible pathway antagonists, including *c-myc, dickkopf 1 (dkk1), dkk2, naked cuticle homologs 1–3 (nkd1–3), notum 1, R-spondin 2 (rspo2), axin2, myc, apc downregulated 1 (apcdd1), wnt1 inducible signaling pathway protein 1 (wisp1), wnt inhibitory factor 1 (wif1), sp5, engrailed2 (en2)*, and *twist1*, was robustly increased in apc^mcr^ mutant larvae, as expected. The visual inspection of this list also revealed an increase in mRNA abundance for many inflammatory cytokines, cytokine receptors, and mediators of cytokine signaling, including *ccl19a, interleukin 11 (il11), leptin, parathyroid hormone 1, suppressor of cytokine signaling 3 (socs3), cxcl19, angiopoietin 2, colony stimulatory factor 1 (csf1)*, and others (Supplemental Table S1). To validate changes in RNA abundance observed by RNA-seq, we performed RT-qPCR on selected genes and found close correlation using the two methods for all genes tested (Figure 3B). These data confirm the increased expression of cytokines, Wnt target genes, and Wnt antagonists. In contrast, the expression of gsc was reduced by greater than five-fold, consistent with the marked reduction in gsc expression observed by in situ hybridization (Figure 2D).

To identify the patterns of gene expression changes in *apc^mcr^* mutants, we performed gene ontology (GO) analysis using Metascape [37]. In addition to confirming the altered expression of Wnt target genes and inflammatory cytokines, this analysis revealed significant changes in the expression of genes related to cell migration, extracellular matrix organization, cell fate specification, and cartilage development (Figure 3C), consistent with alterations in CNC migration, differentiation, and craniofacial development. The reduced expression at 48 hpf of genes enriched in cartilage, including *foxl1*, *loxl2b*, *and2*, *tfap2b*, and *col11a2* (Supplemental Table S1), is consistent with the loss of CNC-derived craniofacial cartilages in *apc^mcr^* mutant larvae.

### 3.3. Increased Expression of Complement c3 in apc^mcr/mcr^ Larvae

Mayor and colleagues showed that the complement C3 cleavage product C3a mediates the mutual attraction of CNC cells when they are migrating as a group and that the overexpression of C3a misdirects and impairs collective CNC cell migration. Consistent with the hypothesis that the CNC defect we observe could be due to disrupted CNC migration, we find the significantly elevated expression of *c3* genes in *apc^mcr^* larvae at 48 hpf (Figure 3D,E). While Carmona-Fontaine et al. examined CNC migration at an earlier stage following delamination [11], it is likely that C3a is also required for collective CNC cell migration at later stages and thus elevated *c3* expression at later stages may also interfere with CNC migration into the head. Gene set enrichment analysis (GSEA [41]) identified the upregulation of additional genes associated with the complement pathway and EMT (Figure 3F), as well as c-Myc, Wnt/β-catenin, and mTORC1 signaling pathways, as expected with an *apc* loss of function mutation (Supplemental Table S3).

These findings are consistent with the hypothesis that the migration of CNC cells into the head is disrupted in *apc* mutants by the overexpression of *c3* genes. However, we also observe changes in the expression of other secreted factors that have been shown to function as CNC chemoattractants or repellents. For example, the expression of *stromal-derived Factor 1 (sdf1)/cxcl12*, which is expressed in pharyngeal endoderm [46] and is required for CNC migration into the head in zebrafish, *Xenopus*, and other organisms [16,46,47], is reduced 2.5-fold in *apc^mcr^* mutant larvae. In addition, multiple semaphorin family genes, axonal guidance molecules that can also instruct the direction of NC migration, show significantly altered expression in *apc^mcr^* larvae (Figure 3D). It should also be pointed out that the RNA-seq analysis was performed at 48 hpf, within the window of CNC differentiation, whereas CNC migration primarily occurs at earlier stages.

### 3.4. An Oncogenic apc Mutation Alters mRNA Splicing

**Figure 4:**
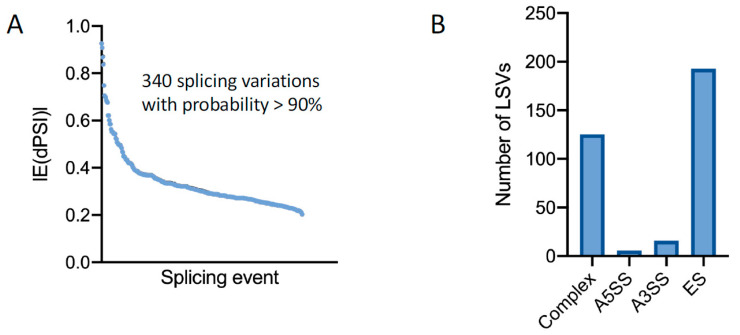

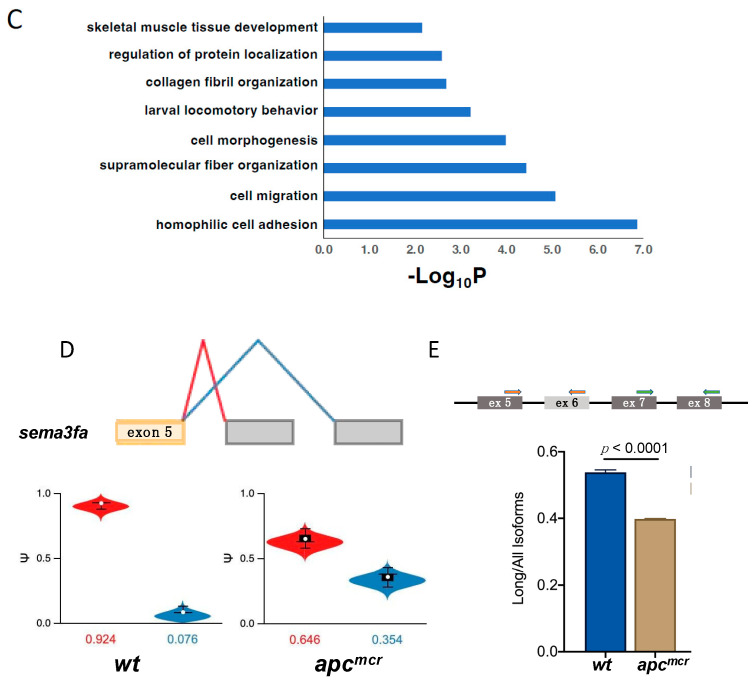
Oncogenic mutations in *apc* cause widespread splice variations including mRNAs encoding regulators of cell migration: (**A**) The MAJIQ analysis of splicing variations, represented as changes in fractions spliced in (dPSI) for each splicing event filtered for dPSI ≥ 0.20 with a probability >0.90, identified 340 splicing variations in *apc^mcr^* larvae. (**B**) The frequency of local splice variants, including exon skipping (ES), alternative 3′ splice site (A3SS), alternative 5′ splice site (A5SS), and complex splice variants. (**C**) The GO analysis of alternative spliced genes. (**D**) Voila output showing alternative splicing of *sema3f* (“a” allele). Red line indicates splicing from exon 5 to exon 6 and blue line indicates splicing from exon 5 to exon 7. Violin plots show the frequency of each splice form in WT (**left**) and *apc^mcr^* mutants (**right**) at 48 hpf. (**E**). Diagram showing unspliced, nascent mRNA from exon 5–exon 8. Orange arrows indicate PCR primers for “long” splice form that includes exon 6; green arrows show the primer pair for constitutively spliced exons 7 and 8, used to measure all mRNA isoforms. Histogram shows relative abundance based on RT-qPCR for the “long” splice form containing exon 6 in WT and *apc^mcr^* mutant larvae at 48 hpf. *p* < 0.0001 (Student’s paired *t*-test, *n* = 3 replicates).

APC enhances the activity of GSK-3, and therefore, GSK-3 activity is reduced by oncogenic *apc* mutations, including the *apc^mcr^* mutation in zebrafish [26,28]. The inhibition of GSK-3 reduces the phosphorylation of multiple splicing factors in diverse mammalian cell types and alters the splicing of hundreds of mRNAs in mammalian cells [35,48,49,50]. Germline mutations in splicing factors are associated with craniofacial defects in humans and mice [51,52]. As somatic mutations in splicing factors are also associated with multiple solid and hematologic malignancies [53,54], we hypothesized that the reduced GSK-3 activity associated with oncogenic *apc* mutations might also disrupt mRNA splicing. We therefore analyzed the above RNA-seq dataset for splicing changes in *apc^mcr^* mutant zebrafish using the MAJIQ pipeline [55]. For a fractional change in splicing (change in percent spliced in or ∆PSI) ≥0.20 with a probability >0.90, we identified 340 splicing variations in *apc^mcr^* larvae compared to wild-type larvae (Figure 4A, Supplemental Table S2). Alternative splicing events included exon skipping, alternative 5′ and alternative 3′ splice sites, and other splicing changes (Figure 4B). GO analysis identified splicing variations in mRNAs encoding regulators of cell adhesion, cell migration, cytoskeleton, and cell morphogenesis (Figure 4C).

We also observed the significantly altered splicing of the “a” allele of *semaphorin 3f* (*sema3fa*; Figure 4D,E), a secreted axonal guidance molecule that is required for CNC migration in mice and chickens [56,57]. Under control conditions, *sema3fa* mRNA predominantly includes exon 6 with less than 8% of transcripts alternatively spliced to skip this exon. In *apc^mcr^* mutants, 35.4% of transcripts skip exon 6, deleting a sequence within the highly conserved sema domain and generating a transcript predicted to encode a nonfunctional protein. Although we have not tested whether these changes have functional consequences, the data nevertheless make it clear that oncogenic mutations in *apc* can markedly alter the splicing of multiple mRNAs across the transcriptome, including mRNAs known to regulate CNC migration.

## 4. Discussion

NC comprises a unique and complex set of multipotent cells that undergo induction, migration, invasion, and differentiation into diverse cell types. Roberto Mayor and his colleagues have made seminal contributions to our understanding of NC induction and migration [1,7,8,9,10,11,12,13,14,15,16,17,18]. Their elucidation of collective cell movements during CNC migration and the role of C3 in mediating the co-attraction of migrating CNC cells suggests the hypothesis that the marked reduction in CNC cells in the head of *apc^mcr/mcr^* larvae could be due to elevated *c3* expression disrupting or misdirecting the migration of CNC cells that normally populate the first and second pharyngeal arches. However, we also observe robust changes in the expression of other factors that guide CNC migration and that could contribute to the CNC defect in *apc^mcr^* zebrafish. Importantly, our data do not rule out the possibility that the defect is primarily due to a change in CNC cell fate specification, despite the normal expression of pan-NC markers *sox10* and *crestin*. Similarly, the reduced expression of *sdf1* raises the possibility that the defects in the mandibular and hyoid arches is caused by altered development of pharyngeal endoderm [46]. While future research will need to examine CNC cells during the migration phase of NC development, the morphological data reported here nevertheless define a novel and dramatic CNC phenotype caused by an *apc* loss of function mutation. Furthermore, our transcriptomic data suggest testable hypotheses for the role of secreted molecules, such as C3, Sdf1, and Sema3f, as downstream targets of APC in the regulation of CNC migration.

The striking absence of *gsc* expression in the mandibular and hyoid arches of *apc^mcr^* larvae is reminiscent of the human neurocristopathy SAMS (short stature, auditory-canal atresia, mandibular hypoplasia, and skeletal abnormalities) that has been associated with *GSC* mutations [58] and is consistent with knockout data in mice showing that *gsc* is required for craniofacial development [59,60].

In humans, *APC* is a classical tumor suppressor gene that is mutated in most cases of colorectal carcinomas. Although neoplasia associated with *APC* mutation is generally ascribed to the activation of canonical Wnt/β-catenin target genes, APC suppresses other downstream targets, and these are also activated by an *APC* loss of function mutations. As APC directly enhances GSK-3 activity, the loss of *APC* reduces GSK-3 activity and activates downstream effectors that are normally inhibited by GSK-3, such as mTORC1. Indeed, multiple *apc^mcr^* phenotypes are reversed by inhibiting mTORC1 [26]. Thus, the CNC/craniofacial defect we observe with *apc* loss may also arise through a GSK-3-dependent but β-catenin-independent mechanism. In support of this hypothesis, conditional KO of *Gsk3* in mice causes pronounced craniofacial defects independently of β-catenin [61].

GSK-3 phosphorylates multiple splicing factors and regulates mRNA splicing at a genome-wide level [35,48,49,50]. Thus, we predicted that loss of *apc* would also disrupt splicing by impairing GSK-3 activity. Indeed, the *apc^mcr/mcr^* mutation significantly alters the usage of 340 splicing variations. Alternatively spliced mRNAs included *sema3f*, which has previously been shown to mediate CNC migration [56] and other genes involved in cell migration. The broad disruption of alternative splicing in *apc^mcr^* mutants suggests a parallel with a group of disorders referred to as craniofacial spliceosomopathies [51,52,62]. Alternative splicing is tightly regulated spatially and temporally during craniofacial development in the mouse in a manner that correlates with the changes in the expression of RNA-binding proteins [62]. Furthermore, multiple human disorders in craniofacial development are associated with germline mutations in splicing factors, spliceosome assembly factors, and other RNA-binding proteins, including *PUF60*, *EFTUD2*, *SF3B4*, *SNRPB*, *SNRPA*, *EIF4A3*, *TXNL4A*, *RBM8A*, *HNRNPR*, *RBM10*, and *HRNNPH2* [51,52,62,63,64]. Thus, the defect in craniofacial development in *apc^mcr^* mutant zebrafish could similarly arise through the disruption of alternative splicing regulated by GSK-3. Future studies will address whether *apc*-dependent splicing variations are mediated through GSK-3 and whether the alternative splicing of *sema3f* alters CNC migration or otherwise contributes to the craniofacial phenotype in *apc^mcr^* mutants. We also suggest the hypothesis that alternative splicing caused by the *APC* loss of function could contribute to the malignant phenotypes associated with *APC*-related cancers.

NC migration has been frequently compared to the metastasis of epithelial cancers [6,17,18,20]. In both cases, the cells exit their initial locations through EMT mediated by the increased expression of *Snail* family transcription factors and the down-regulation of epithelial cell adhesion molecules, such as E-Cadherin. Upon delamination, CNC cells undergo collective cell migration, as described by Mayor’s group, in a manner that is similar to more recent observations in pancreatic, colorectal, and breast cancers, in which tumor cells undergo “partial EMT” (p-EMT) and then migrate collectively as cell clusters before invading their final destinations [65,66,67,68]. Similar to CNC, ovarian cancer cells secrete C3, which is induced in these cells by the EMT factor Twist1 and is expressed at the invasive edge of metastatic cells in vivo [69]. We observe the increased expression of *twist1* in the *apc^mcr^* mutant larvae, and this could also contribute to the increase in *c3a* expression. Thus, C3 could serve a similar function in APC-related cancers by maintaining the association of metastatic cells in transit.

## 5. Conclusions

In summary, we report that a truncating mutation in the tumor suppressor gene *apc* in zebrafish disrupts CNC-dependent craniofacial development. The deep sequencing of expressed RNAs reveals the increased expression of complement *c3* in *apc* mutant larvae. Based on the seminal work of Mayor and colleagues, showing that C3 overexpression impairs CNC migration, we propose the testable hypothesis that the overexpression of *c3* caused by the loss of *apc* disrupts or misdirects CNC migration into the head. However, the *apc^mcr^* mutation also alters the expression of other important mediators of CNC migration, including the reduced expression of *SDF1/CXCL12* and varied effects on multiple semaphorin family members. These changes in expression or altered cell fate not identified by sox10 or crestin expression analysis could alternatively be responsible for the marked craniofacial defects we observe in *apc^mcr^* larvae. Finally, we show that Apc significantly regulates the splicing of hundreds of mRNAs, including *sema3f*. We propose that this is through the direct modulation of GSK-3 activity and that disrupted splicing associated with *APC* loss of function mutations may contribute to craniofacial defects during zebrafish development as well as the pathogenesis of colon cancer in humans. These data provide a basis for future studies into the roles of APC, GSK-3, and mRNA splicing in cell migration and fate specification in normal development and in cancer metastasis.

## Figures and Tables

**Table 1:** Primers for reverse transcription-quantitative PCR (RT-qPCR).

RT-qPCR	Forward	Reverse
CCL19	CCCCATTGCAGCTACTGTATTC	AGGTGTTTTTCTCTGGTGGGG
IL-11a	CCGGTTCAAGTCTCTTCCAG	AGGTTTGCATGGAGCTGAGA
IL-11b	CATCTTATCCAAGCTATCATCCAG	GATCTCGGGTGCTGTCTGTC
LEPA	GGAACACATTGACGGGCAAA	ATGGGTTTGTCAGCGGGAAT
CBX7a	TGCGGTGGAGTCAATAACGAA	CAGGTGCTGTACTTAGGCGA
DKK1a	GTGGAGTTTGTCTGTCGTGC	AAGCTGACACACACCGTTGA
DKK1b	CACCGCAGCAGCCTTTAATC	CGCGAGACTGGAAGCAAAAC
NOTUM1a	CACCTGTAACGACGGGACTC	CCAGCCGCCCTCAAGAAATA
LFT1	ACGCACGAGTGAGCATCTAC	CGTGAATGGGAATCAACCTGG
SALL1b	CTTCAGGGAGATAACCCGGC	CCATCGTATGATCGAGGAGAACA
SOCS3a	AAGCAGGGAAGACAAGAGCC	AGAGCTGGTCAAAAGAGCCTAT
GSC	CCTACAGGTTATGACAGCGCC	GACAAGGTGCCCACGTTCAT
EMILIN3a	GGCACAAGAACCACTGTGCAT	CCAAGCACACTTCATTGCCT
GAPDH	GTGGAGTCTACTGGTGTCTTC	GTGCAGGAGGCATTGCTTACA
C3A.1	GACGCCCAACTTGAAACCAC	GCAACCTCAGGGATGGCATA
C3A.6	ACCAGGAATGCCCTTCAGTG	ACCTTTGACTCCTCCGGGAT
RT-qPCR for splice forms:		
SEMA3fa_ALL	ACTCAACAGCGTCTCAGCTT	TACTGGTCGGTCCTCATTGC
SEMA3fa_Long splice form	ACAACCCCATCTGCACCTATG	TCTGGTGAGGCACTAGGGT

## Data Availability

RNA-seq data were deposited in the NCBI Gene Expression Omnibus [70] and are accessible through GEO Series accession number GSE234986. (https://www.ncbi.nlm.nih.gov/geo/query/acc.cgi?acc=GSE234986).

## Supplementary Materials

The following supporting information can be downloaded at: https://www.mdpi.com/article/10.3390/jdb11030029/s1, Supplemental Table S1: RNA-seq apc vs. wt; Supplemental Table S2: splicing analysis; Supplemental Table S3: GSEA.

## References

[1] Aybar M.J., Mayor R. (2002). Early induction of neural crest cells: Lessons learned from frog, fish and chick. Curr. Opin. Genet. Dev..

[2] Jain R., Rentschler S., Epstein J.A. (2010). Notch and cardiac outflow tract development. Ann. N. Y. Acad. Sci..

[3] LaBonne C., Bronner-Fraser M. (2000). Snail-related transcriptional repressors are required in Xenopus for both the induction of the neural crest and its subsequent migration. Dev. Biol..

[4] Mayor R., Theveneau E. (2013). The neural crest. Development.

[5] Shellard A., Mayor R. (2016). Chemotaxis during neural crest migration. Semin. Cell Dev. Biol..

[6] Klymkowsky M.W., Rossi C.C., Artinger K.B. (2010). Mechanisms driving neural crest induction and migration in the zebrafish and Xenopus laevis. Cell Adh. Migr..

[7] Bajanca F., Gouignard N., Colle C., Parsons M., Mayor R., Theveneau E. (2019). In vivo topology converts competition for cell-matrix adhesion into directional migration. Nat. Commun..

[8] Barriga E.H., Franze K., Charras G., Mayor R. (2018). Tissue stiffening coordinates morphogenesis by triggering collective cell migration in vivo. Nature.

[9] Canales Coutino B., Mayor R. (2021). The mechanosensitive channel Piezo1 cooperates with semaphorins to control neural crest migration. Development.

[10] Carmona-Fontaine C., Matthews H.K., Kuriyama S., Moreno M., Dunn G.A., Parsons M., Stern C.D., Mayor R. (2008). Contact inhibition of locomotion in vivo controls neural crest directional migration. Nature.

[11] Carmona-Fontaine C., Theveneau E., Tzekou A., Tada M., Woods M., Page K.M., Parsons M., Lambris J.D., Mayor R. (2011). Complement fragment C3a controls mutual cell attraction during collective cell migration. Dev. Cell.

[12] Mancilla A., Mayor R. (1996). Neural crest formation in Xenopus laevis: Mechanisms of Xslug induction. Dev. Biol..

[13] Rabadan M.A., Herrera A., Fanlo L., Usieto S., Carmona-Fontaine C., Barriga E.H., Mayor R., Pons S., Marti E. (2016). Delamination of neural crest cells requires transient and reversible Wnt inhibition mediated by Dact1/2. Development.

[14] Shellard A., Mayor R. (2021). Collective durotaxis along a self-generated stiffness gradient in vivo. Nature.

[15] Shellard A., Szabo A., Trepat X., Mayor R. (2018). Supracellular contraction at the rear of neural crest cell groups drives collective chemotaxis. Science.

[16] Theveneau E., Marchant L., Kuriyama S., Gull M., Moepps B., Parsons M., Mayor R. (2010). Collective chemotaxis requires contact-dependent cell polarity. Dev. Cell.

[17] Szabo A., Mayor R. (2018). Mechanisms of Neural Crest Migration. Annu. Rev. Genet..

[18] Theveneau E., Mayor R. (2012). Neural crest delamination and migration: From epithelium-to-mesenchyme transition to collective cell migration. Dev. Biol..

[19] Theveneau E., Duband J.L., Altabef M. (2007). Ets-1 confers cranial features on neural crest delamination. PLoS ONE.

[20] Nieto M.A. (2013). Epithelial plasticity: A common theme in embryonic and cancer cells. Science.

[21] Abercrombie M., Heaysman J.E. (1953). Observations on the social behaviour of cells in tissue culture. I. Speed of movement of chick heart fibroblasts in relation to their mutual contacts. Exp. Cell Res..

[22] Ji Y., Hao H., Reynolds K., McMahon M., Zhou C.J. (2019). Wnt Signaling in Neural Crest Ontogenesis and Oncogenesis. Cells.

[23] Hutchins E.J., Bronner M.E. (2018). Draxin acts as a molecular rheostat of canonical Wnt signaling to control cranial neural crest EMT. J. Cell Biol..

[24] Maj E., Kunneke L., Loresch E., Grund A., Melchert J., Pieler T., Aspelmeier T., Borchers A. (2016). Controlled levels of canonical Wnt signaling are required for neural crest migration. Dev. Biol..

[25] Shull L.C., Lencer E.S., Kim H.M., Goyama S., Kurokawa M., Costello J.C., Jones K., Artinger K.B. (2022). PRDM paralogs antagonistically balance Wnt/beta-catenin activity during craniofacial chondrocyte differentiation. Development.

[26] Valvezan A.J., Huang J., Lengner C.J., Pack M., Klein P.S. (2014). Oncogenic mutations in adenomatous polyposis coli (Apc) activate mechanistic target of rapamycin complex 1 (mTORC1) in mice and zebrafish. Dis. Model. Mech..

[27] Valvezan A.J., Klein P.S. (2012). GSK-3 and Wnt Signaling in Neurogenesis and Bipolar Disorder. Front. Mol. Neurosci..

[28] Valvezan A.J., Zhang F., Diehl J.A., Klein P.S. (2012). Adenomatous polyposis coli (APC) regulates multiple signaling pathways by enhancing glycogen synthase kinase-3 (GSK-3) activity. J. Biol. Chem..

[29] Ji L., Lu B., Wang Z., Yang Z., Reece-Hoyes J., Russ C., Xu W., Cong F. (2018). Identification of ICAT as an APC Inhibitor, Revealing Wnt-Dependent Inhibition of APC-Axin Interaction. Mol. Cell.

[30] Tran H., Polakis P. (2012). Reversible modification of adenomatous polyposis coli (APC) with K63-linked polyubiquitin regulates the assembly and activity of the beta-catenin destruction complex. J. Biol. Chem..

[31] Hurlstone A.F., Haramis A.P., Wienholds E., Begthel H., Korving J., Van Eeden F., Cuppen E., Zivkovic D., Plasterk R.H., Clevers H. (2003). The Wnt/beta-catenin pathway regulates cardiac valve formation. Nature.

[32] Goessling W., North T.E., Lord A.M., Ceol C., Lee S., Weidinger G., Bourque C., Strijbosch R., Haramis A.P., Puder M. (2008). APC mutant zebrafish uncover a changing temporal requirement for wnt signaling in liver development. Dev. Biol..

[33] Westerfield M. (1993). The Zebrafish Book a Guide for the Laboratory Use of Zebrafish Danio (Brachydanio) Rerio.

[34] Thisse C., Thisse B. (2008). High-resolution in situ hybridization to whole-mount zebrafish embryos. Nat. Protoc..

[35] Shinde M.Y., Sidoli S., Kulej K., Mallory M.J., Radens C.M., Reicherter A.L., Myers R.L., Barash Y., Lynch K.W., Garcia B.A. (2017). Phosphoproteomics reveals that glycogen synthase kinase-3 phosphorylates multiple splicing factors and is associated with alternative splicing. J. Biol. Chem..

[36] Nguyen-McCarty M., Klein P.S. (2017). Autophagy is a signature of a signaling network that maintains hematopoietic stem cells. PLoS ONE.

[37] Zhou Y., Zhou B., Pache L., Chang M., Khodabakhshi A.H., Tanaseichuk O., Benner C., Chanda S.K. (2019). Metascape provides a biologist-oriented resource for the analysis of systems-level datasets. Nat. Commun..

[38] Durinck S., Spellman P.T., Birney E., Huber W. (2009). Mapping identifiers for the integration of genomic datasets with the R/Bioconductor package biomaRt. Nat. Protoc..

[39] Cunningham F., Allen J.E., Allen J., Alvarez-Jarreta J., Amode M.R., Armean I.M., Austine-Orimoloye O., Azov A.G., Barnes I., Bennett R. (2022). Ensembl 2022. Nucleic Acids Res..

[40] Durinck S., Moreau Y., Kasprzyk A., Davis S., De Moor B., Brazma A., Huber W. (2005). BioMart and Bioconductor: A powerful link between biological databases and microarray data analysis. Bioinformatics.

[41] Subramanian A., Tamayo P., Mootha V.K., Mukherjee S., Ebert B.L., Gillette M.A., Paulovich A., Pomeroy S.L., Golub T.R., Lander E.S. (2005). Gene set enrichment analysis: A knowledge-based approach for interpreting genome-wide expression profiles. Proc. Natl. Acad. Sci. USA.

[42] Mootha V.K., Lindgren C.M., Eriksson K.F., Subramanian A., Sihag S., Lehar J., Puigserver P., Carlsson E., Ridderstrale M., Laurila E. (2003). PGC-1alpha-responsive genes involved in oxidative phosphorylation are coordinately downregulated in human diabetes. Nat. Genet..

[43] Lawson N.D., Weinstein B.M. (2002). In vivo imaging of embryonic vascular development using transgenic zebrafish. Dev. Biol..

[44] Schulte-Merker S., Hammerschmidt M., Beuchle D., Cho K.W., De Robertis E.M., Nusslein-Volhard C. (1994). Expression of zebrafish goosecoid and no tail gene products in wild-type and mutant no tail embryos. Development.

[45] Nusse R. (1999). The Wnt Gene Homepage. http://www.stanford.edu/~rnusse/wntwindow.html.

[46] Olesnicky Killian E.C., Birkholz D.A., Artinger K.B. (2009). A role for chemokine signaling in neural crest cell migration and craniofacial development. Dev. Biol..

[47] Belmadani A., Tran P.B., Ren D., Assimacopoulos S., Grove E.A., Miller R.J. (2005). The chemokine stromal cell-derived factor-1 regulates the migration of sensory neuron progenitors. J. Neurosci..

[48] Heyd F., Lynch K.W. (2010). Phosphorylation-dependent regulation of PSF by GSK3 controls CD45 alternative splicing. Mol. Cell.

[49] Hernandez F., Perez M., Lucas J.J., Mata A.M., Bhat R., Avila J. (2004). Glycogen synthase kinase-3 plays a crucial role in tau exon 10 splicing and intranuclear distribution of SC35. Implications for Alzheimer’s disease. J. Biol. Chem..

[50] Wang S.B., Venkatraman V., Crowgey E.L., Liu T., Fu Z., Holewinski R., Ranek M., Kass D.A., O’Rourke B., Van Eyk J.E. (2018). Protein S-Nitrosylation Controls Glycogen Synthase Kinase 3beta Function Independent of Its Phosphorylation State. Circ. Res..

[51] Beauchamp M.C., Alam S.S., Kumar S., Jerome-Majewska L.A. (2020). Spliceosomopathies and neurocristopathies: Two sides of the same coin?. Dev. Dyn..

[52] Griffin C., Saint-Jeannet J.P. (2020). Spliceosomopathies: Diseases and mechanisms. Dev. Dyn..

[53] Kandoth C., McLellan M.D., Vandin F., Ye K., Niu B., Lu C., Xie M., Zhang Q., McMichael J.F., Wyczalkowski M.A. (2013). Mutational landscape and significance across 12 major cancer types. Nature.

[54] Cherry S., Lynch K.W. (2020). Alternative splicing and cancer: Insights, opportunities, and challenges from an expanding view of the transcriptome. Genes Dev..

[55] Vaquero-Garcia J., Barrera A., Gazzara M.R., Gonzalez-Vallinas J., Lahens N.F., Hogenesch J.B., Lynch K.W., Barash Y. (2016). A new view of transcriptome complexity and regulation through the lens of local splicing variations. Elife.

[56] Gammill L.S., Gonzalez C., Bronner-Fraser M. (2007). Neuropilin 2/semaphorin 3F signaling is essential for cranial neural crest migration and trigeminal ganglion condensation. Dev. Neurobiol..

[57] Osborne N.J., Begbie J., Chilton J.K., Schmidt H., Eickholt B.J. (2005). Semaphorin/neuropilin signaling influences the positioning of migratory neural crest cells within the hindbrain region of the chick. Dev. Dyn..

[58] Parry D.A., Logan C.V., Stegmann A.P., Abdelhamed Z.A., Calder A., Khan S., Bonthron D.T., Clowes V., Sheridan E., Ghali N. (2013). SAMS, a syndrome of short stature, auditory-canal atresia, mandibular hypoplasia, and skeletal abnormalities is a unique neurocristopathy caused by mutations in Goosecoid. Am. J. Hum. Genet..

[59] Rivera-Perez J.A., Mallo M., Gendron-Maguire M., Gridley T., Behringer R.R. (1995). Goosecoid is not an essential component of the mouse gastrula organizer but is required for craniofacial and rib development. Development.

[60] Yamada G., Mansouri A., Torres M., Stuart E.T., Blum M., Schultz M., De Robertis E.M., Gruss P. (1995). Targeted mutation of the murine goosecoid gene results in craniofacial defects and neonatal death. Development.

[61] Gonzalez Malagon S.G., Lopez Munoz A.M., Doro D., Bolger T.G., Poon E., Tucker E.R., Adel Al-Lami H., Krause M., Phiel C.J., Chesler L. (2018). Glycogen synthase kinase 3 controls migration of the neural crest lineage in mouse and Xenopus. Nat. Commun..

[62] Hooper J.E., Jones K.L., Smith F.J., Williams T., Li H. (2020). An Alternative Splicing Program for Mouse Craniofacial Development. Front. Physiol..

[63] Bain J.M., Cho M.T., Telegrafi A., Wilson A., Brooks S., Botti C., Gowans G., Autullo L.A., Krishnamurthy V., Willing M.C. (2016). Variants in HNRNPH2 on the X Chromosome Are Associated with a Neurodevelopmental Disorder in Females. Am. J. Hum. Genet..

[64] Marques F., Tenney J., Duran I., Martin J., Nevarez L., Pogue R., Krakow D., Cohn D.H., Li B. (2016). Altered mRNA Splicing, Chondrocyte Gene Expression and Abnormal Skeletal Development due to SF3B4 Mutations in Rodriguez Acrofacial Dysostosis. PLoS Genet..

[65] Aiello N.M., Maddipati R., Norgard R.J., Balli D., Li J., Yuan S., Yamazoe T., Black T., Sahmoud A., Furth E.E. (2018). EMT Subtype Influences Epithelial Plasticity and Mode of Cell Migration. Dev. Cell.

[66] Cheung K.J., Ewald A.J. (2016). A collective route to metastasis: Seeding by tumor cell clusters. Science.

[67] Friedl P., Locker J., Sahai E., Segall J.E. (2012). Classifying collective cancer cell invasion. Nat. Cell Biol..

[68] Simeonov K.P., Byrns C.N., Clark M.L., Norgard R.J., Martin B., Stanger B.Z., Shendure J., McKenna A., Lengner C.J. (2021). Single-cell lineage tracing of metastatic cancer reveals selection of hybrid EMT states. Cancer Cell.

[69] Cho M.S., Rupaimoole R., Choi H.J., Noh K., Chen J., Hu Q., Sood A.K., Afshar-Kharghan V. (2016). Complement Component 3 Is Regulated by TWIST1 and Mediates Epithelial-Mesenchymal Transition. J. Immunol..

[70] Edgar R., Domrachev M., Lash A.E. (2002). Gene Expression Omnibus: NCBI gene expression and hybridization array data repository. Nucleic Acids Res..

